# An Amine-Reactive Phenazine Ethosulfate (arPES)—A Novel Redox Probe for Electrochemical Aptamer-Based Sensor

**DOI:** 10.3390/s22051760

**Published:** 2022-02-24

**Authors:** Madoka Nagata, Jinhee Lee, Stephen Henley, Kazunori Ikebukuro, Koji Sode

**Affiliations:** 1 Joint Department of Biomedical Engineering, The University of North Carolina at Chapel Hill and North Carolina State University, Chapel Hill, NC 27599, USA; mnagata@email.unc.edu (M.N.); jinc0324@gmail.com (J.L.); sdhenley7@gmail.com (S.H.); 2 Department of Biotechnology and Life Science, Graduate School of Engineering, Tokyo University of Agriculture and Technology, Koganei 184-8588, Japan; ikebu@cc.tuat.ac.jp

**Keywords:** aptamer, redox probe, amine-reactive phenazine ethosulfate, electrochemical sensor

## Abstract

Electrochemical aptamer-based biosensors (E-ABs) are attractive candidates for use in biomarker detection systems due to their sensitivity, rapid response, and design flexibility. There are only several redox probes that were employed previously for this application, and a combination of redox probes affords some advantages in target detection. Thus, it would be advantageous to study new redox probes in an E-AB system. In this study, we report the use of amine-reactive phenazine ethosulfate (arPES) for E-AB through its conjugation to the terminus of thrombin-binding aptamer. The constructed E-AB can detect thrombin by square-wave voltammetry (SWV), showing peak current at −0.15 V vs. Ag/AgCl at pH 7, which differs from redox probes used previously for E-ABs. We also compared the characteristics of PES as a redox probe for E-AB to methylene blue (MB), which is widely used. arPES showed stable signal at physiological pH. Moreover, the pH profile of arPES modified thrombin-binding aptamer revealed the potential application of arPES for a simultaneous multianalyte detection system. This could be achieved using different aptamers with several redox probes in tandem that harbor various electrochemical peak potentials. Our findings present a great opportunity to improve the current standard of biological fluid monitoring using E-AB.

## 1. Introduction

Aptamers are single-stranded oligonucleotides that bind to their target with affinity and specificity and is widely used as biosensing molecules for biosensors [1,2]. Because of the flexibility of their structure, electrochemical sensors using aptamers were reported based on the detection of conformation change of aptamers during the recognition of target molecules, denoted as electrochemical aptamer-based biosensors, or E-ABs [3]. A widely used approach for creating E-ABs is to modify the target aptamer at one terminus with a redox probe while using the other terminus to immobilize the aptamer on the electrode. Conformational change induced by target binding thus changes the orientation of the redox probe and alters its accessibility relative to the electrode surface. This consequently alters the kinetics of electron transfer between the redox-probe and electrode, and this change can be interrogated with a variety of electrochemical techniques (e.g., square-wave voltammetry and differential scanning voltammetry). As a result, the detection and monitoring of target concentration can be observed as a function of change in faradaic current without bound/free separation. To date, this principle was successfully utilized for continuous monitoring in vivo of target molecules such as nucleic acids, proteins, and small molecules [4,5,6].

The most representative redox probe used for aptamer modification is methylene blue (MB) [5,7]. The modification of aptamers by MB is often achieved through the conjugation reaction of N-hydroxysuccinimide ester (NHS ester)-modified MB with an amine-terminated oligonucleotide sequence [8]. Although the use of other redox probes was reported in this application, such as ferrocene [9,10] or anthraquinone [11], there are limitations associated with their availabilities.

**Scheme 1:**
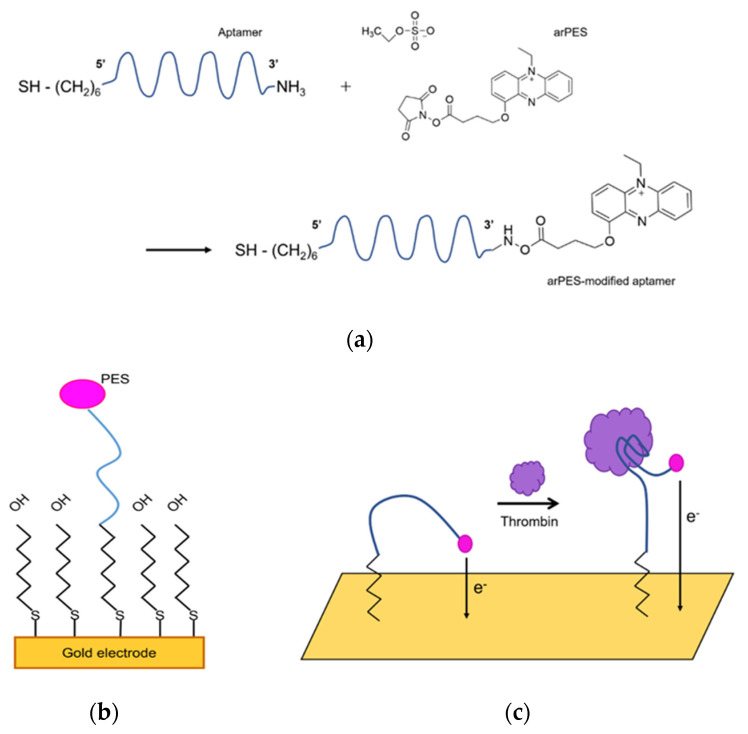
(**a**) Schematic detailing conjugation of arPES to 3′-amine of aptamer by succinimidyl-ester aminolysis. (**b**) Schematic diagram of arPES-modified aptamer immobilized to electrode surface. (**c**) Schematic detailing conformational change of arPES-modified aptamer upon binding to thrombin. Conformational change of aptamer induced by target-binding changes electron transfer kinetics between PES and electrode, which can be monitored by voltammetry.

We previously reported a novel redox probe suitable for modification of protein amines, N-hydroxysuccinimidylester 1-propoxy-5-ethylphenazinium ethyl sulfate, or amine-reactive phenazine ethosulfate (arPES) (Scheme 1a). The modification of redox enzymes by arPES can be achieved by a rapid and single-step conjugation, through the reaction of the N-hydroxysuccinimide ester moiety of arPES with solvent-accessible primary amine groups (e.g., Lysine residues). Thus, conjugation of arPES enabled the preparation of quasi-direct electron transfer type enzymes that do not require a mediator in solution [12,13,14,15]. Recently, we also reported the construction of a continuous glutamine sensor using arPES-modified glutamine binding protein, which undergoes a conformation change upon interaction with glutamine [16]. This study revealed that arPES is able to be used to monitor the dynamic change of biomolecules continuously. Considering its availability and versatility, arPES is a redox probe that could be suitable for the preparation of E-ABs.

In this study, we report the preparation of an arPES-modified thrombin-binding aptamer (TBA), and this construct’s application for the development of an E-AB (Scheme 1b). We chose TBA [17] as a representative aptamer because it exhibits stark conformation change upon the binding of its target molecule (thrombin) and is extremely well characterized for E-AB [5]. We investigated the electrochemical properties of arPES-modified TBA and its application for thrombin detection (Scheme 1c). When investigating the effect of pH, the arPES-modified aptamer demonstrated less pH sensitivity compared with that of MB-modified TBA.

## 2. Materials and Methods

### 2.1. Chemical and Materials

The oligonucleotide (5′ SH-(CH_2_)_6_-TAAGTTCATCTCCCCGGTTGGTGTGGTTGGT-NH_3_ 3′) was purchased from IDT (Coralville, IA, USA).

Sodium chloride, potassium chloride, sodium phosphate dibasic, potassium phosphate monobasic, 6-mercapto-1-hexanol, tricine, tris(2-carboxyethyl)phosphine (TCEP) and sodium acetate were purchased from Sigma–Aldrich Co. LLC (St. Louis, MO, USA). Purchased from Fisher Scientific Co. LLC (Pittsburgh, PA, USA) were N-Cyclohexyl-2-aminoethanesulfonic acid (CHES), 2-(N-morpholino)ethanesulfonic acid (MES), 2-amino-2-(hydroxymethyl)propane-1,3-diol (Tris) and human alpha thrombin. Amine reactive phenazine ethosulfate (arPES) was kindly donated from Dojindo Molecular Technology, Inc. (Rockville, MD, USA). Purchased from Dojindo Molecular Technology, Inc. (Rockville, MD, USA) was 1-methoxy-phenazine ethosulfate (mPES). Sodium hydroxide, sulfuric acid, and ethanol were purchased from VWR (Radnor, PA, USA). Platinum wire was purchased from TANAKA Kikinzoku (Tokyo, Japan). Silver/silver chloride (3 M NaCl) reference electrodes were purchased from BAS Inc. (Tokyo, Japan). Gold disk electrodes were purchased from CH Instrumental, Inc. (Austin, TX, USA).

### 2.2. Modification of Amine-Reactive PES (arPES) to Thrombin Binding Aptamer

Amine-modified thrombin-binding aptamer (TBA) (100 µM) was mixed with arPES in 400 mM tricine buffer (pH 8.0) and incubated at 25 °C overnight. After incubation, arPES-modified TBA was purified from the mixture by ethanol precipitation.

### 2.3. Preparation of Aptamer-Immobilized Gold Electrode

Bare gold electrodes were prepared by polishing with alumina powder, sonication in water and absolute ethanol. Polished electrodes were cleaned electrochemically [18]. Cleaned gold electrodes were dipped into 200 mM Tris-HCl buffer (pH 7.4) containing 10 µM TCEP and 1 µM arPES-TBA overnight. After this step, electrodes immobilized with arPES-modified TBA were immersed into a blocking solution containing 1 mM 6-MCH into 200 mM Tris-HCl buffer (pH 7.4) for 2 h. Fabricated electrodes were soaked into 10 mM phosphate-buffered saline (137 mM NaCl, 2.7 mM KCl, 10 mM Na_2_HPO_4_, 1.8 mM KH_2_PO_4_, pH 7.4) (PBS) buffer before measurement.

### 2.4. Electrochemical Measurement of Thrombin Concentration Using arPES-Modified TBA

All the electrochemical measurements were performed using the VersaSTAT4 potentiostat (Princeton Applied Research, Princeton, NJ, USA). Fabricated gold electrodes immobilized with arPES-modified TBA were interrogated from −0.1 to −0.4 V (vs. Ag/AgCl) with 25 mV amplitude, 4 mV steps and 5 to 250 Hz frequency range. Following the measurement of square-wave voltammetry (SWV) at 0 M thrombin, each concentration of thrombin solution was added to the electrochemical analysis cells. After the incubation for 30 min, thrombin concentration dependency was measured by SWV.

### 2.5. SWV Measurement in Several pH Conditions

Fabricated sensors were interrogated from −0.1 to −0.4 V (vs. Ag/AgCl) with 25 mV amplitude, 4 mV steps, and 5 to 250 Hz frequency range. SWV measurements were carried out in several pH buffer solutions. pH 6.0; 50 mM MES buffer, pH 7.0 and pH 8.0; 50 mM Tris buffer, pH 9.0, and pH 10.0; 50 mM CHES buffer.

## 3. Results

### 3.1. Electrochemical Characterization of arPES-Modified TBA Aptamer Immobilized Electrode

**Figure 1:**
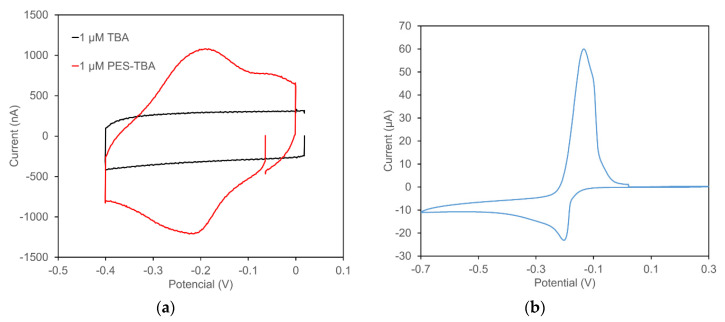
Cyclic voltammograms of thrombin binding aptamer (TBA) with or without modification by amine-reactive phenazine ethosulfate (arPES) (**a**), cyclic voltammogram of free methoxy-PES (mPES). (**b**) Cyclic voltammetry measurements were performed in 5 mL of PBS buffer (pH 7.4) in the presence of 1 µM TBA or PES modified (**a**) or 1 mM mPES (**b**), respectively. Scan rate was 600 mV/s (**a**), or 20 mV/s (**b**), respectively.

The results of cyclic voltammetry (CV) measurements are shown in Figure 1. The black line in Figure 1a is the cyclic voltammogram for the aptamer immobilized on the electrode without PES modification, and the red line is for the immobilized PES-modified aptamer. Both cyclic voltammograms were obtained from measurement in PBS buffer solution (pH 7.4). PES-modified aptamers clearly show redox peaks at around −0.2 V (Figure 1a), which was identical to the result observed in cyclic voltammogram for free mPES (Figure 1b). This peak was not observed from the electrode immobilized with TBA without arPES modification (Figure 1a). The difference in the peak height and shapes between electrode immobilized PES-TBA and free mPES are due to the difference in the concentrations of redox probes and localization of redox probe. The total amount of PES on the electrode with PES-modified TBA was less than 50 pmol, which was calculated from the PES-TBA solution, whereas Figure 1b shows the CV of the solution containing 1 mM mPES, thereby showing this significant difference in the peak height. The oxidation and reduction potentials of the electrode with PES-modified TBA were −0.19 V and −0.20 V, respectively. Since PES-modified TBA was immobilized on the electrode surface, the diffusion of the redox probe would be negligible. Consequently, the peak current separation derived from the electrode with PES-modified TBA was smaller than it of free mPES (the oxidation and reduction potentials were −0.13 V and −0.20 V, respectively). These results demonstrate that TBA was successively modified with arPES, and PES can serve as a redox probe when conjugated to aptamers on the electrode surface.

### 3.2. Electrochemical Measurement of Thrombin Using PES-Modified TBA Immobilized Electrode

**Figure 2:**
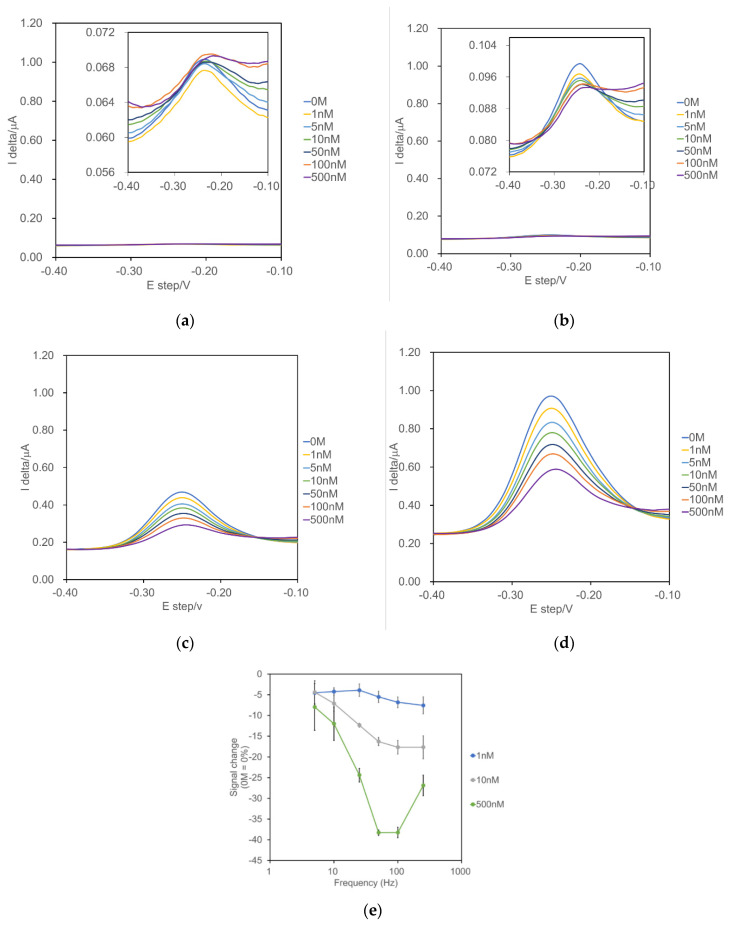
Representative square-wave voltammograms of fabricated arPES-modified thrombin aptamer immobilized electrodes at (**a**) 5 Hz, (**b**) 10 Hz, (**c**) 50 Hz, and (**d**) 100 Hz, and peak current change dependencies on frequency at each thrombin concentration (**e**). Square-wave voltammetry (SWV) scans were conducted as following conditions: −0.4 to −0.1 V, 25 mV amplitude. Seven concentrations of thrombin were used: 0, 1, 5, 10, 50, 100, and 500 nM. Insets in (**a**,**b**) show enlarged voltammograms.

Figure 2a–d show representative square-wave voltammograms of electrodes immobilized with PES-modified TBA in the presence of different concentrations of thrombin (1–500 nM) and different frequencies (5–250 Hz). Peak current changes derived from PES were observed with all investigated frequencies that decreased in concentration-dependent thrombin. These observations were consistent with the expectation that thrombin binding would trigger a change of TBA conformation, which consequently affected the electron transfer dynamics between PES and electrode. This was reported previously using MB as a redox probe [5]. However, the observed peak currents at lower frequencies (5 Hz and 10 Hz) were so low compared with the ones observed at higher frequencies (50 Hz and 100 Hz), that the base line changes at the lower frequency observations were not negligible compared with that of the thrombin concentration-dependent peak current change, thereby making the measurement difficult (the insets of Figure 2a,b). To obtain the highest signal gain, the optimal frequency of thrombin measurement was investigated. With this sensing principle, the frequency of SWV strongly affects signal gain [19]. Since fabricated electrodes showed a signal off response from up to 10 Hz and maximum peak current change was shown at 100 Hz (Figure 2e), we decided to use 100 Hz as the optimal frequency for thrombin monitoring using electrodes immobilized with PES-modified TBA.

**Figure 3:**
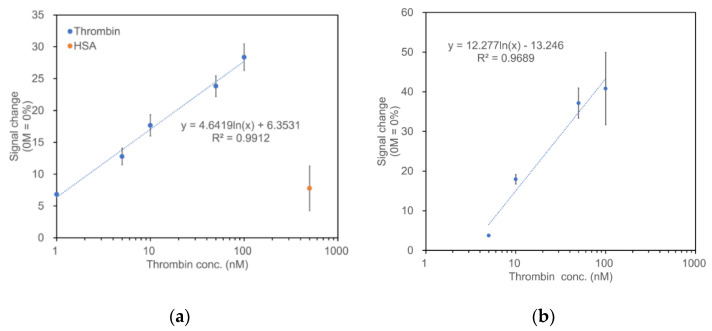
Thrombin concentration-dependent signal change (%) at PES-modified TBA immobilized electrodes (**a**) and at MB-modified TBA immobilized electrodes (**b**). Calculated as 0 M = 0%. Error bars indicate standard deviations (*n* = 3).

Figure 3a shows the correlation of peak current change rate and logarithmic values of thrombin concentration with a dynamic range of 1–100 nM. A good linear correlation between the sensor signal and thrombin concentration (logarithmic) was observed in addition to the electrode with MB-TBA (Figure 3b). On the contrary, the addition of 500 nM human serum albumin (HSA) provided a slight peak current change, which corresponded to the peak current change obtained at 1 nM thrombin, providing evidence for the selectivity of the constructed EAB for thrombin.

The limit of detection (LOD) was calculated as 3SD/slope, where SD is the standard deviation of the peak current of blank and the slope is from the calibration curve. The obtained LOD was 14.7 nM. These results indicate that arPES can be used as a redox probe to construct electrochemical aptamer-based biosensors.

### 3.3. Effect of pH on Thrombin Sensor Employing PES Modified TBA

**Figure 4:**
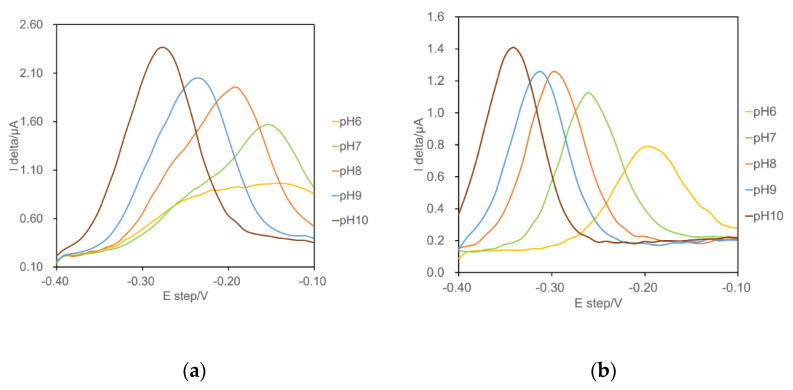
Square-wave voltammograms of an arPES-TBA immobilized electrode (**a**) and a methylene blue (MB)-TBA immobilized electrode (**b**) under several pH conditions.

To investigate the effect of pH on the sensor response of PES modified TBA immobilized electrode, and its comparison with MB modified TBA one, SWV measurements were carried out in several pH solutions. Representative results are shown in Figure 4. The peak potential of electrodes immobilized with PES-modified TBA was shifted negatively dependent on the pH increase, from −0.15 V (pH 7) to −0.27 V (pH 10) vs. Ag/AgCl (Figure 4a). On the other hand, the peak potential of electrodes immobilized with MB-modified TBA was also shifted from −0.25 V (pH 7) to −0.33 V (pH 10) vs. Ag/AgCl (Figure 4b).

## 4. Discussion

To provide an alternative redox probe for electrochemical aptamer sensor development, the novel redox probe, arPES, was investigated through its conjugation to TBA, combined with gold electrodes and analysis by SWV. TBA is a well-studied aptamer, and it was employed previously as a signal-off E-AB [5]. The constructed electrodes with PES-modified TBA showed a peak current decrease which was dependent on thrombin concentration, showing the potential of arPES to be used for an alternative redox probe for E-AB development.

The stability of methoxy-PES, the structure of arPES after its conjugation to the aptamer via amine coupling reaction, was previously investigated in solution. It was revealed that this redox probe is stable for 30 days at 30 °C within a pH range of 4 to 8, keeping 90% of initial activity [20]. Additionally, biosensors using arPES were able to detect their analyte from biologically relevant, complex sample matrices, such as artificial cerebrospinal fluids (aCSF) [21]. The aCSF contains a higher salt concentration than PBS buffer. The previous report was carried out using aCSF by adding human serum albumin to mimic real CSF, revealing that arPES-modified biosensing molecule works within a relevant biological fluid. Additionally, the detection of digested haemoglobin A1c using arPES-modified enzyme was also reported [15]. These reports indicate the feasibility of arPES application in the biological samples within a complex sample matrix for E-ABs.

For SWV, the measured current is correlated with the electron transfer rate between redox probe and the electrode, relative to the frequency of the square-wave pulse. For example, if the frequency is set as low with the redox probe which shows the rapid electron transfer rate, rapid electron transfer reaction decayed before sampling the current, and as a result, the obtained signal is diminished [19]. Therefore, we attempted to optimize for the appropriate detection frequency.

**Figure 5:**
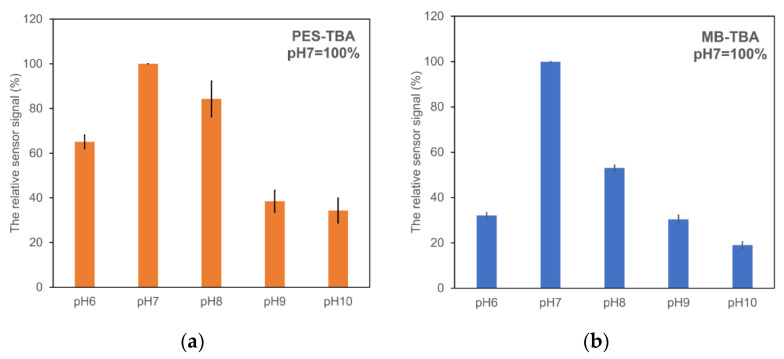
pH dependency of sensor signal observed at peak potential normalized to pH 7 with electrodes immobilized with PES-modified TBA (**a**) and electrodes immobilized with MB-modified TBA (**b**). Sensor signals in solution at pH 7, −0.15 V vs. Ag/AgCl for PES-modified TBA, and −0.25 V vs. Ag/AgCl for MB-modified TBA, are plotted against rest of range (pH 6–10) as 100%.

One of the inherent issues in the translation of redox probe-modified aptamers for sensing application is the impact of pH on sensor response [22]. Considering the potential application of E-ABs for monitoring disease, health, the environment, and process validation, methods for broadening the pH range applicable for sensing should be investigated. In this study, we investigated the pH dependency of sensors immobilized with arPES-modified TBA and compared its response to sensors immobilized with MB-modified TBA. Figure 5 summarizes these results by comparing the sensor signal observed at peak potential at pH 7; −0.15 V vs. Ag/AgCl for the electrode with PES-modified TBA, and −0.25 V vs. Ag/AgCl for the electrode with MB-modified TBA, respectively. The electrode with PES-modified TBA maintained 80% of the current at pH 8 (Figure 5a), whereas the electrode with MB-modified TBA decreased to 60% at pH 8 (Figure 5b), indicating the electrode immobilized with PES-modified TBA showed less pH sensitivity in the physiological pH compared with that of the MB-modified one. These observations suggest that arPES is a versatile redox probe, tolerant under a wide range of pH conditions, and they also indicate its feasibility when applied as a redox probe for E-ABs.

Furthermore, these results revealed the possibility of utilizing arPES in combination with other aptamers modified by redox probe, such as MB, anthraquinone, and/or ferrocene, since their peak potentials are different from arPES. With SWV measurement in PBS buffer (pH 7.4), electrodes immobilized with PES-modified TBA showed peak current at −0.25 V, whereas electrodes immobilized with MB-modified TBA showed peak current at −0.3 V (data not shown). The difference of redox peak potentials remained constant across several pH solutions (Figure 4a,b). Previously, a signal-on/off sensing platform with multiredox probes was reported for the detection of thrombin with high sensitivity [23]. Additionally, it was reported that the combination of these redox probes with different aptamers enabled a multiplexed detection of several different targets [24,25,26] as well as helped to correct baseline drift during continuous measurement in serum [27]. The use of arPES as an additional redox probe in E-ABs, as shown promisingly in this study, could facilitate advances in this type of multiplexed E-AB design, improving the capacity for biological fluid monitoring via an E-AB platform.

## 5. Conclusions

In this work, we demonstrated the use of phenazine ethosulfate (PES)-modified thrombin-binding aptamer (TBA) in the construction of a signal-off electrochemical aptamer-based biosensor (E-AB). We succeeded in detecting thrombin through SWV measurement using electrodes immobilized with the PES-modified TBA over a thrombin concentration range of 1–100 nM. Our sensor showed a peak current at −0.25 V vs. Ag/AgCl, which was different from previously reported redox probes used in this application, MB and ferrocene. Moreover, we confirmed that electrodes immobilized with PES-modified TBA showed stable peak current at physiological pH. These observations demonstrated the potential utility of amine-reactive phenazine ethosulfate (arPES) as a component of a simultaneous multianalyte detection system. In such a system, several aptamers could be conjugated with different redox probes each with their own unique peak potential. Considering the design of future E-ABs, this finding poses a great advantage for biological fluid monitoring.

## Data Availability

The data presented in this study are available in the insert article.
